# The Fungus *Nosema ceranae* and a Sublethal Dose of the Neonicotinoid Insecticide Thiamethoxam Differentially Affected the Health and Immunity of Africanized Honey Bees

**DOI:** 10.3390/microorganisms11051258

**Published:** 2023-05-10

**Authors:** Alvaro De la Mora, Nuria Morfin, José C. Tapia-Rivera, José O. Macías-Macías, José M. Tapia-González, Francisca Contreras-Escareño, Tatiana Petukhova, Ernesto Guzman-Novoa

**Affiliations:** 1 School of Environmental Sciences, University of Guelph, Guelph, ON N1G2W1, Canada; delamora@uoguelph.ca (A.D.l.M.); nmorfinr@uoguelph.ca (N.M.); 2 Centro de Investigaciones en Abejas, CUSUR, Universidad de Guadalajara, Enrique Arreola Silva 883, Zapotlan el Grande 49000, Jalisco, Mexico; jose.tapia@cusur.udg.mx (J.C.T.-R.); joseoc@cusur.udg.mx (J.O.M.-M.); joset@cusur.udg.mx (J.M.T.-G.); 3 Departamento de Producción Agricola, CUCSUR, Universidad de Guadalajara, Independencia Nal. 161, Autlan 48900, Jalisco, Mexico; franciscacon@cucsur.udg.mx; 4 Department of Population Medicine, University of Guelph, Guelph, ON N1G2W1, Canada; tpetukho@uoguelph.ca

**Keywords:** neonicotinoid insecticides, *Nosema ceranae*, *Apis mellifera*, haemocytes, cellular immunity, humoral immunity, gene expression

## Abstract

Honey bees (*Apis mellifera* L.) are affected by different biotic and abiotic stressors, such as the fungus *Nosema ceranae* and neonicotinoid insecticides, that negatively impact their health. However, most studies so far conducted have focused on the effect of these stressors separately and in European honey bees. Therefore, this study was conducted to analyze the impact of both stressors, singly and in combination, on honey bees of African descent that have demonstrated resistance to parasites and pesticides. Africanized honey bees (AHBs, *Apis mellifera scutellata* Lepeletier) were inoculated with *N. ceranae* (1 × 10^5^ spores/bee) and/or chronically exposed for 18 days to a sublethal dose of thiamethoxam (0.025 ng/bee) to evaluate their single and combined effects on food consumption, survivorship, *N. ceranae* infection, and immunity at the cellular and humoral levels. No significant effects by any of the stressors were found for food consumption. However, thiamethoxam was the main stressor associated to a significant decrease in AHB survivorship, whereas *N. ceranae* was the main stressor affecting their humoral immune response by upregulating the expression of the gene *AmHym-1*. Additionally, both stressors, separately and combined, significantly decreased the concentration of haemocytes in the haemolymph of the bees. These findings indicate that *N. ceranae* and thiamethoxam differentially affect the lifespan and immunity of AHBs and do not seem to have synergistic effects when AHBs are simultaneously exposed to both stressors.

## 1. Introduction

Honey bees (*Apis mellifera* L.) are the most important pollinators of agricultural crops and are valuable contributors to the pollination of wild plants [1]. This is why it is concerning that beekeepers in North America and other parts of the world have been experiencing an unusually high loss of honey bee colonies during the last decade [2,3]. The causes of these high colony mortality rates are most likely related to the interaction of multiple stressing factors. Two of the most common stressors honey bees are exposed to are the fungus *Nosema ceranae* and neonicotinoid insecticides [4].

*Nosema ceranae* is a spore-forming fungus of the Class Microsporidia that infects the midgut epithelial cells of adult honey bees [5], and that is now widely distributed in many countries [6,7,8,9]. Although the evidence is not conclusive, *N. ceranae* infection has been identified as one of the stressors that may be associated with colony losses in North America and parts of Europe [10,11,12]. The infection by *N. ceranae* negatively affects nutrient digestion and absorption, causing energetic stress in the bees [13,14,15]. *Nosema ceranae* infected bees have a reduced lifespan compared to non-infected bees [16,17,18]. Moreover, *N. ceranae* can affect metabolic and immune pathways [19], impacting innate defense mechanisms by apparently immunosuppressing its host [20,21,22].

Neonicotinoids are neurotoxic systemic insecticides that act as acetylcholine receptor agonists in the central nervous system of insects [23]. Among these insecticides, thiamethoxam is one of the most widely used worldwide and is commonly applied as seed treatment of corn, soybean, and sunflower [24]. Bees are repeatedly exposed to sublethal doses of neonicotinoids while frequently collecting nectar and/or pollen from plants grown from pesticide-treated seeds [25]. Although a few studies have found no deleterious effects of neonicotinoids on honey bee health at the colony level [26,27], sublethal exposure to neonicotinoids has been reported to affect learning and memory [28,29], disrupt foraging activities [30,31,32], cause immune changes [33,34], affect the development of hypopharyngeal glands [35], decrease survivorship, and activate detoxification mechanisms in honey bees [36,37].

Studies on the combined effects of *N. ceranae* and neonicotinoids have found synergistic effects on increased honey bee mortality [38,39,40], but no interactions on haemocyte counts, phenoloxidase activity [38], or detoxification mechanisms [39]. Thus, the interaction between sublethal exposure to neonicotinoids and *N. ceranae* infections seems to be complex, and most of these types of studies have been conducted using honey bees of European subspecies (EHBs). Honey bees of tropical origin, such as the so-called Africanized bees (AHBs, descendants of *A. m. scutellata* Lepeletier), have demonstrated resistance to parasites and viruses [41,42,43,44], and higher tolerance to several insecticides in comparison with EHBs [45,46]. Moreover, no studies have investigated the effect of *N. ceranae* infection and/or exposure to neonicotinoid insecticides on both, the cellular and humoral immune responses of honey bees of African descent. Therefore, the objective of this study was to assess the effect of chronic exposure to thiamethoxam and/or *N. ceranae* on survivorship, infection development, and immune responses of AHBs.

## 2. Materials and Methods

### 2.1. Ethical Statement

This study was conducted under the supervision of researchers of the Bee Research Center, University of Guadalajara, Zapotlan, JAL, Mexico, and the Honey Bee Research Centre, University of Guelph, Guelph, ON, Canada. No permits were required to conduct the study.

### 2.2. Sources of Honey Bees and Nosema Ceranae Spores

The honey bees used in this study were obtained from three colonies located at the Bee Research Center, University of Guadalajara, in Zapotlan, Jalisco, Mexico (19°43′31″ N, 103°27′41″ W). Their Africanized origin was corroborated by analyzing samples for mtDNA and morphometric type [47] at the Honey Bee Research Centre, University of Guelph, in Canada. Bees obtained from the three experimental colonies were equally represented in all treatment groups.

For spore source and extraction, forager bees were collected from a colony previously identified to have high infection levels of *N. ceranae* [48]. A survey previously conducted by our group to identify pathogens of honey bees in the study area detected only *N. ceranae* but no *N. apis* (unpublished data). The guts of 20–30 bees were dissected and macerated in ddH_2_O to extract and purify spores of the parasite as per McGowan et al. [49]. Briefly, the macerate was filtered and centrifuged at 800× *g* for 8 min three times. The resulting pellet was re-suspended in ddH_2_O and the spores of *Nosema* in the suspension were quantified with a haemocytometer [48]. The average of three counts was used to calculate the number of spores per µL in the suspension, which was used to obtain a final suspension of 1 × 10^5^ spores/µL of ddH_2_0 through serial dilutions. The identification of only *N. ceranae* was verified in several subsamples as per Hamiduzzaman et al. [50].

### 2.3. Thiamethoxam Dilutions

A field realistic concentration of thiamethoxam was calculated based on the knowledge that a honey bee consumes 25–39 mg of nectar per day [51] and that the concentration of neonicotinoids in nectar from canola-treated plants range between 0.001 and 0.009 ng/mg [52,53]. Thus, the amount of thiamethoxam that a bee could consume in a day would be between 0.025 and 0.351 ng, which is about 13 to 176 times lower than the 24 h LD_50_ (4.4 ng) for honey bees [54]. The lowest amount of this range (0.025 ng) was used to calculate a thiamethoxam concentration of 1 × 10^−3^ ng/µL of food, considering an average consumption of 25 µL of sucrose syrup per bee per day [28,55]. To prepare the working dilutions, 10 mg of thiamethoxam (Sigma Aldrich^®^, Oakville, ON, Canada) were diluted in 100 mL of ddH_2_O and subsequently serially diluted to 10 ng/mL.

### 2.4. Treatments

Newly emerged bees (<24 h) were obtained from frames with capped brood incubated in screened emerging cages (5 × 28 × 25 cm) at 35 °C and 60% RH overnight. A subsample of 30 randomly chosen workers was used to confirm that they were free of *Nosema* spores [48]. Thirty bees were randomly assigned to each of four treatments and were starved for 2 h before being treated. The treatments were 1 × 10^5^ *N. ceranae* spores/bee (N), 1 × 10^−3^ ng/µL-thiamethoxam (T), 1 × 10^5^ *N. ceranae* spores/bee + 1 × 10^−3^ ng/µL-thiamethoxam (NT), and the control (C) that was not exposed to thiamethoxam, or to *N. ceranae*, and only received 50% sucrose syrup. The *N. ceranae* dose (1 × 10^5^ spores/bee) was chosen to ensure infection development in the inoculated bees as previously demonstrated [49].

For the two *N. ceranae* treatments (N and NT), 10 µL of the spore suspension (1 × 10^5^ spores/µL of ddH_2_0) were diluted in 90 µL of 50% sucrose syrup to obtain a suspension of 1 × 10^4^ spores/µL. Each bee was fed individually with 10 µL of the treated syrup (1 × 10^5^ spores per bee) using a micropipette (Eppendorf, Mississauga, ON, Canada). The bees of treatments C and N were fed with 50% sucrose syrup only, whereas those of treatments T and NT received the experimental sublethal dose of thiamethoxam in the syrup for 18 days to emulate field realistic conditions. To prepare the syrup with the desired concentration of the pesticide, 10 mL of thiamethoxam solution (10 ng/mL) were diluted in 90 mL of 50% sucrose syrup.

Bees were kept in sterilized hoarding cages (15 × 10 × 10 cm) inside an incubator at 35 °C and 60% RH for 18 days, because it is known that the life cycle of *N. ceranae* lasts five to seven days, so spores could replicate more than one cycle. The bees were provided with dH_2_O and 50% sucrose syrup (containing or not thiamethoxam) ad libitum in gravity feeders. The syrup was replaced every day, and consumption was calculated daily by weighing the feeders before and 24 h after placing them on the cages, using a balance (Denver Instruments S-403, Bohemia, NY, USA). To obtain the amount of syrup consumed per bee, data were adjusted for daily mortality by dividing the amount of syrup consumed by the number of live bees in the cage. Dead bees were counted and removed every day. After 18 days, the remaining live bees were collected and kept at −70 °C until further analysis. This experiment was replicated six times.

### 2.5. Nosema Ceranae Infectivity and Infection Intensity

For the two groups of bees treated with *N. ceranae* spores (N and NT) and the control (C), workers were individually dissected and microscopically analyzed to determine whether they had detectable *N. ceranae* spores [48] to obtain infectivity rates for the parasite. Then, the individual samples were pooled for spore counts to determine *N. ceranae* infection intensity [48]. No *N. ceranae* spores were detected in bees from treatment C. The sample size for these analyses varied between 36 and 90 bees per treatment, depending on the treatment.

### 2.6. Effect of Stressors on Cellular Immunity

The effect of *N. ceranae* infection and thiamethoxam exposure on cellular immunity was assessed by estimating the concentration of haemocytes in the haemolymph of experimental bees. Between six and twelve bees from each experimental group were randomly selected at the end of the experiment and before freezing them to collect a sample of 4 µL haemolymph from each of them. Briefly, each bee was pierced with an entomological pin between the second and third dorsal tergite and the sample of haemolymph was collected with a micropipette. The haemolymph was spread over a microscope slide and stained with Hema 3^®^ (Fisher Health Care Protocol, Mississauga, ON, Canada). The number of haemocytes per µL of haemolymph of the sampled bees was obtained as per Koleoglu et al. [56], and six replicates of these assessments were conducted.

### 2.7. Effect of Stressors on Humoral Immunity

The effect of the two stressors on humoral immunity was evaluated by measuring the expression of the immune-related gene *hymenoptaecin* (*AmHym-1*, GB51223). Hymenoptaecin, the product of *AmHym-1,* is an antimicrobial peptide (AMP) [57] that has been widely used as a marker to evaluate humoral immunity in honey bees [58]. The extraction of RNA and gene expression analysis of the samples were conducted at the Honey Bee Research Centre, University of Guelph, in Canada. Total RNA was extracted from three bee abdomens pooled from each biological repetition using TRIzol^®^ Reagent (Fisher Scientific, Mississauga, ON, Canada) as per the manufacturer’s instructions. The RNA concentration (ng/µL) and quality values (absorbance ratio, 260/280 nm = 1.8−2.0) were determined with a spectrophotometer (NanodropLite™, Thermo Scientific, Mississauga, ON, Canada).

cDNA was prepared using a RevertAid™ H Minus First Strand cDNA Synthesis Kit (Fermentas, Burlington ON, Canada) following the manufacturer’s instructions, with 2000 ng of RNA per sample. The cDNA was stored at −20 °C until used for gene expression analysis. The primers reported by Evans [59] were used for the target gene, whereas beta actin (*β-actin*, GB44311) was used as reference gene, and was amplified with the primers used by Di Prisco et al. [60].

The efficiencies of the target and reference genes were determined based on standard curves of known concentration using 10-fold serial dilutions (10^9^−10^1^ copies) of 300 bp synthetic gene fragments (gBlocks^®^; Integrated DNA Technologies, Coralville, IA, USA). The qRT-PCR was performed with a BioRad CFX96™ thermocycler (Bio-Rad Laboratories, Mississauga, ON, Canada) with PowerUp™ SYBR Master Mix (2X) (Thermo Fisher Scientific, Mississauga, ON, Canada). Reactions were conducted in 20 µL: 2 µL of template, 0.6 µL of each primer (300 nM), 10 µL of PowerUp™ SYBR Master Mix (2X), and 6.8 µL of nuclease free H_2_O. The cycling protocol consisted of one cycle at 50 °C for 2 min, one at 95 °C for 10 min, and then 40 cycles at 95 °C for 15 s, and 60 °C for 60 s.

The expression level of the target gene was normalized to the expression level of the reference gene using the 2^−ΔΔ^ (Livak) method [61] with the control group as calibrator. The Bio-Rad CFX Manager^®^ 3.1 software (Bio-Rad Laboratories, Mississauga, ON, Canada) was used to calculate the expression ratio.

### 2.8. Statistical Analyses

The data on sucrose syrup consumption was square root transformed before subjecting them to a one-way ANOVA as it did not comply with normality based on a Shapiro–Wilk test. The data on proportion of infected bees were analyzed using the Welch two sample test. The data on *N. ceranae* intensity were analyzed using the Wilcoxon test, as they could not be normalized. The data on number of haemocytes and *AmHym-1* gene expression were tested with a Shapiro–Wilk test and due to lack of normality, were natural-log and log_2_ transformed, respectively, before being analyzed with a one-way ANOVA and Fisher LSD tests. The survivorship data were subjected to survival analysis using the Kaplan–Meier log rank test, and the curves were compared using the Holm correction for multiple comparisons. The above statistical analyses were performed using R 3.3.1 (Foundation for Statistical Computing, Vienna, Austria).

## 3. Results

### 3.1. Effect of Nosema Ceranae And/or Thiamethoxam on Food Consumption and Survivorship

**Figure 1:**
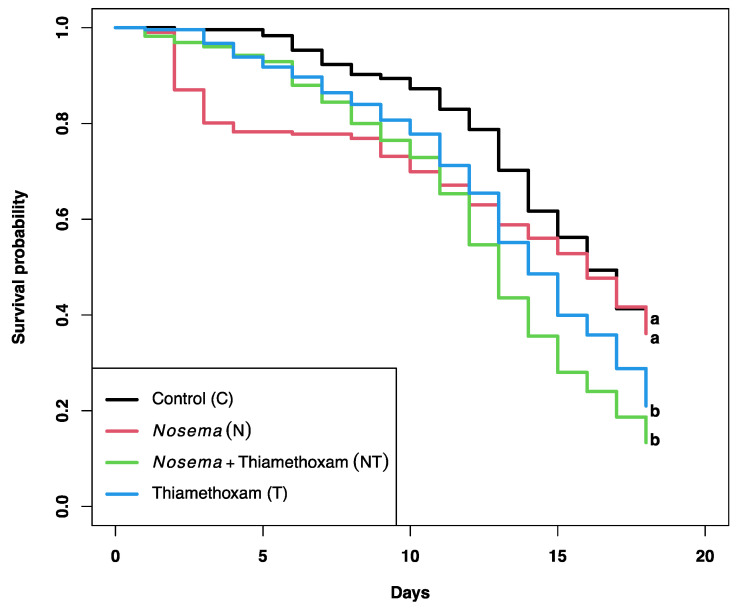
Kaplan–Meier curves showing the survival probability of honey bees exposed to a sublethal dose of thiamethoxam (1 × 10^−3^ ng/µL of syrup) and/or *Nosema ceranae* (1 × 10^5^ spores per bee) for 18 consecutive days. Log rank and Holm correction for multiple comparisons were used to determine significant differences between curves, which are indicated by different literals.

The mean daily sucrose syrup consumption per bee was not significantly different between treatments (F_3, 428_ = 1.376, *p*= 0.250; Table 1). However, a significant reduction in the probability of bee survival was found for some treatments (*X*^2^ = 50.5, df = 3, *p* < 0.0001; Figure 1). Pairwise comparisons showed a significant decrease in the proportion of bees that survived between treatments C (0.37) and T (0.13; *p* < 0.001), and between treatments C and NT (0.21, *p* < 0.01). However, no significant differences in honey bee survival were observed between treatments C and N (0.36; *p* = 0.844), showing that the main stressor associated with a decrease in bee survivorship was thiamethoxam.

### 3.2. Effect of Thiamethoxam on Nosema Ceranae Infectivity and Infection Intensity

Thiamethoxam did not affect the infectivity of *N. ceranae* as the mean proportion of infected bees in treatment N was not significantly different from that of bees in treatment NT (t = −0.24, *p* = 0.8; Table 2). For infection intensity, the number of replicated *N. ceranae* spores after 18 days of inoculation in N and NT treated bees was 43 and 13 times higher than the initial inoculum, respectively. However, despite this large gap in the number of spores between treatments, there were no significant differences in spore counts between N and NT-treated bees (W = 1242, *p* = 0.47; Table 2).

### 3.3. Effect of Nosema Ceranae and/or Thiamethoxam on Cellular Immunity

**Figure 2:**
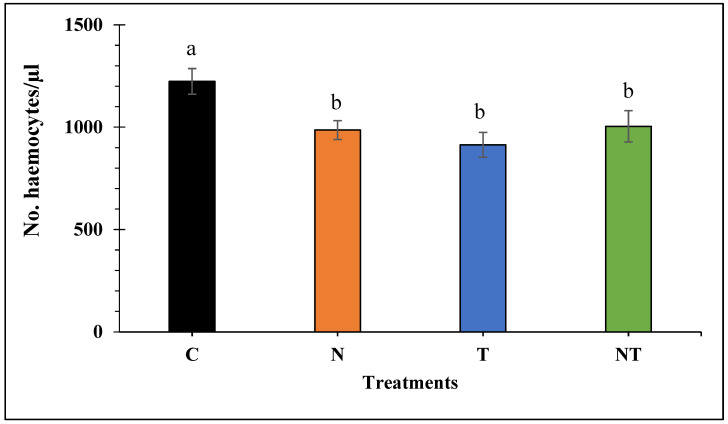
Mean number of haemocytes per µL of haemolymph of honey bees subjected to the treatments for 18 days: Control (C), *Nosema ceranae* (1 × 10^5^ spores per bee; N), sublethal dose of thiamethoxam (1 × 10^−3^ ng/µL of syrup; T), and *N. ceranae* + thiamethoxam (NT). Different literals indicate significant differences between treatments based on one-way ANOVA and Fisher LSD tests of natural log-transformed data. Figure shows untransformed data.

*Nosema ceranae* infection, thiamethoxam exposure, or the combination of both stressors, significantly reduced the concentration of haemocytes in treated bees compared to the control (F_3, 229_ = 5.493, *p* < 0.001; Figure 2). Bees of treatment C had the highest haemocyte counts, whereas bees of treatment T showed the lowest haemocyte counts. However, except for the control, no significant differences were found in haemocyte concentration between the other treatment groups (*p* > 0.05).

### 3.4. Effect of Nosema Ceranae and/or Thiamethoxam on Humoral Immunity

**Figure 3:**
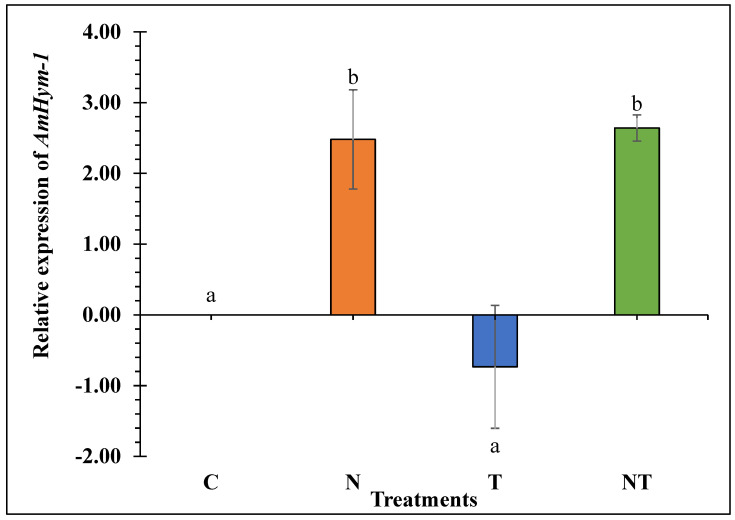
*AmHym-1* relative expression in honey bees subjected to the treatments for 18 days: Control (C), *Nosema ceranae* (1 × 10^5^ spores per bee; N), sublethal dose of thiamethoxam (1 × 10^−3^ ng/µL of syrup; T), *N. ceranae* + thiamethoxam (NT). *AmHym-1* expression was calculated using the Livak 2^−ΔΔCt^ method, with *β-actin* as reference gene and 0 ng as calibrator. Different literals indicate significant differences between treatments based on one-way ANOVA and Fisher LSD tests. Log_2_ transformed data are presented.

*Nosema ceranae* significantly upregulated *AmHym-1* in N and NT-treated bees relative to the control (2.48 log_2_ and 2.64 log_2_ fold up regulation, respectively) (F_3, 11_ = 6.15, *p* = 0.017; Figure 3). Thiamethoxam treated bees did not differ from the control in the expression of this gene. Therefore, *N. ceranae* was the only stressor associated with an over-expression of *AmHym-1*.

## 4. Discussion

Africanized honey bee health and immunity were differentially affected by both *N. ceranae* infection and sublethal thiamethoxam exposure. However, sucrose syrup consumption was not affected by any of the treatments. Other studies have also shown similar results in that *N. ceranae* alone or combined with neonicotinoid insecticides did not affect syrup consumption in experimental bees [15,62,63]. Conversely, Kessler et al. [64] showed that acute oral exposure to thiamethoxam (doses > 25 times higher than the one used in this study) increased the food consumption of treated bees. Thus, the differences between studies seem to be related to the dose used.

Results of this study showed that *N. ceranae* infection did not have a significant effect on AHB survivorship and that thiamethoxam was the main stressor associated with reduced bee survivorship. Our study is in line with findings of previous reports that have also shown a reduction in the lifespan of bees exposed to thiamethoxam at doses several times higher than the one used in this study [65], as well as in combination with *N. ceranae* infection [63,66,67]. However, Gregorc et al. [68] reported no effect of a single exposure of AHBs to thiamethoxam, with doses five to fifty times lower than the one used in this study, and found no effects of *N. ceranae* and no interaction between thiamethoxam and *N. ceranae* on honey bee survivorship. Thus, it appears that the effect of thiamethoxam on honey bee survivorship is related to the dose and time of exposure. We chronically fed the bees with the pesticide for 18 days, which was meant to emulate a realistic field situation.

The decreased survivorship of bees exposed to thiamethoxam in this study may be related to its cytotoxic effect on cells of the central nervous system and on epithelial cells of the bee’s midgut, possibly resulting in an increase in physiological, energetic, and nutritional stress [68,69]. Additionally, the impact of the pesticide on bee lifespan might be related to effects on the cellular immune responses of the exposed bees (see discussion below).

*N. ceranae* infections did not have a significant effect on honey bee survivorship despite the fact that the parasite multiplied more than a dozen times relative to the initial inoculum. This means that the bees were continuously exposed to higher levels of *N. ceranae* infections over time such as chronic exposure to thiamethoxam, but still had less impact on survivorship than the pesticide.

The absence of a significant effect of *N. ceranae* infection on honey bee survivorship in this study could be explained by the activation of humoral immune responses in the experimental bees, including the upregulation of *AmHym-1* [70], for the production of AMPs to neutralize microsporidian infections. These results could also be explained by the relatively short duration of the experiment. The impact of *N. ceranae* infection on honey bee mortality is more apparent after three weeks of infection [15,17,18]. Additionally, it could have been that we used low-pathogenic variants of *N. ceranae* that might not significantly affect honey bee longevity [71].

The proportion of bees infected with *N. ceranae* spores did not differ between N and NT-treated bees. In both groups, the percentage of infected bees was below 50%. A similar percentage of infected bees (<60%) was also reported in workers of AHB colonies from Brazil [72]. However, other studies have reported infectivity rates of 100% in EHBs inoculated with similar or lower concentration of *Nosema* spores than the one used in this study [49,73]. Thus, it seems that AHBs might be more suited to resist the establishment of *N. ceranae* infection compared to EHBs. Nevertheless, further studies are warranted to confirm these results.

*Nosema ceranae* infection intensity at the end of the experiment did not differ significantly between bees of treatment N and bees of treatment NT. The absence of significant differences in spore counts between bees inoculated with *N. ceranae* only and bees that were also exposed to a thiamethoxam sublethal dose has been observed in other studies [63,66]. However, even when thiamethoxam did not show a significant effect on the number of replicated spores in the analysis of this study, the spore loads in bees from group NT were 3.3 times lower compared to those of bees from group N (1.3 vs. 4.4 million spores/bee). The lack of statistical significance between the two experimental groups was probably due to the large variation in infection levels within treatments, which is common for *Nosema* spp. infections in honey bees [49]. These results, even when not significant, suggest that thiamethoxam might inhibit the replication of *N. ceranae* in the bees’ midgut. The inhibitory effect on *N. ceranae* proliferation has been observed for thiamethoxam and other neonicotinoids such as imidacloprid, thiacloprid and fipronil that also decreased *N. ceranae* spore loads in honey bees [38,39,62,68], and in the stingless bee *Melipona colimana* [74]. It is possible that neonicotinoids have antifungal activity as has been documented for imidacloprid that reduced the multiplication of fungi in beetles [75]. Conversely, other studies have reported that neonicotinoid insecticide exposure, including thiamethoxam, increased the number of *N. ceranae* spores in honey bees [39,76,77]. The disagreement of the above studies could be due to factors such as experimental setups, type and dose of neonicotinoid used, time of exposure, disease resistance of the experimental bees used, and genetic variability of *N. ceranae* strains. These hypotheses to explain the above results require further investigation.

The number of *N. ceranae* replicated spores in the inoculated bees of this study was about three to four times lower than what has been reported for EHBs in similar experiments, using the same or lower concentration of *Nosema* spp. inoculum [49,73,78]. These results may again indicate higher resistance of AHBs to *N. ceranae* relative to EHBs as has been suggested by several authors. For example, *N. ceranae* has been infecting AHBs in Brazil for over 40 years, with no evidence of disease or damage to the colonies [7]. Additionally, Mendoza et al. [79] found that bees from AHB colonies had 20% lower spore counts compared to bees of EHB colonies managed under similar conditions, whereas Fleites-Ayil et al. [80] reported low *N. ceranae* infection levels in AHBs compared to those reported from EHB studies. All these studies suggest more resistance to nosema disease in AHBs. However, additional research is needed to support this speculation.

Haemocytes mediate cellular immunity in insects including honey bees, providing defense mechanisms against fungi, bacteria, parasites, and viruses [81,82]. Bees of treatments T, N, and NT showed a significant decrease in haemocyte counts after 18 days of treatment compared to the control, indicating cellular immune suppression by the two stressors. Consequently, it may be possible that cellular immune suppression could potentially increase the bees’ susceptibility to other pathogens.

Such as in this study, suppression of honey bee cellular immunity due to sublethal exposure to neonicotinoids has been documented previously. For example, Brandt et al. [33] observed a decrease in haemocyte concentration, encapsulation response, and melanization in honey bees exposed to sublethal doses of clothianidin, thiacloprid, and imidacloprid. Likewise, *N. ceranae* decreased the number of circulating haemocytes over time in stingless bees [74]. Conversely, other studies reported that sublethal doses of neonicotinoid insecticides, including thiamethoxam and clothianidin, did not affect haemocyte concentration or slightly increased haemocyte counts in the haemolymph of honey bees exposed to the pesticides for 10 or less days of treatment [38,83]. Thus, the effect of thiamethoxam on decreasing the number of haemocytes in the bees of this study could be related to the relatively long-time of exposure to the chemical. In other insects, the number of haemocytes tends to decrease sharply after two weeks [84]. In this study, haemocyte counts were determined 18 days after treatments. Therefore, the lower haemocyte concentration observed in *N. ceranae* infected bees may have occurred over several days during which haemocytes engulfed spores of the parasite [85], thus reducing the number of circulating haemocytes. Another evidence that supports this explanation of our results is that *N. ceranae* infection has been found to reduce the concentration of circulating haemocytes by decreasing the expression of the gene *glucose dehydrogenase* [20], which is involved in the encapsulation of fungal pathogens [86].

The results of this study showed that the combination of *N. ceranae* infection and thiamethoxam exposure decreased the concentration of haemocytes in the haemolymph of honey bees by about the same degree caused by either of the stressors alone, indicating that both stressors separately and combined affected the cellular immune responses of bees similarly, but showed no synergistic effects. In addition to the mechanisms mentioned above to explain how neonicotinoids and *N. ceranae* may affect the immune cellular responses of honey bees, it has been shown that both stressors alter the encapsulation mechanism of haemocytes [87], which could further explain these results. However, this is something that warrants further investigation.

*AmHym-1* regulates the production of hymenoptaecin, which is an important AMP of the immune humoral response in insects, because it has strong antimicrobial activity against bacteria and fungi [40,82]. *Nosema ceranae* was the only stressor that affected the expression of *AmHym-1* in our experimental bees. An increase in the expression of this immune-related gene suggests that the bees reacted to the fungal infection by producing hymenoptaecin as a defense against the microsporidium. Although previous studies have shown that *N. ceranae* may downregulate the expression of *AmHym-1* in bees at an early stage of infection [20,21], this and other recent studies have demonstrated an overexpression of this gene in response to infections by *N. ceranae* at >seven days post inoculation [78,88]. It seems that it takes at least one *N. ceranae* reproductive cycle to activate the production of AMPs for host defense against the fungus. Another study that chronically exposed AHBs to sublethal doses of imidacloprid found that the pesticide downregulated *AmHym-1* [67], but the researchers used doses 100 times higher than the one used in this study.

Experimental bees were exposed to the sublethal dose of thiamethoxam longer than they were to *N. ceranae* infections since it takes at least five days for the microsporidium to complete a life cycle. However, if the pesticide had been more harmful than *Nosema* infection to the bees because of longer initial exposure (first five days), we would have seen that impact for all parameters measured and not only for survivorship when analyzing the effect of the separate treatments (T vs. N). Thiamethoxam did not affect cellular immunity more than *Nosema* infections and did not significantly influence humoral immunity.

## 5. Conclusions

Thiamethoxam was the main stressor associated with a decrease in AHB survivorship, whereas *N. ceranae* was the main stressor affecting their humoral immune response by upregulating the expression of *AmHym-1*. Additionally, both stressors inhibited the cellular immune response of the bees by reducing the concentration of circulating haemocytes, although no synergistic effects were observed. The dose of thiamethoxam and the concentration of spores of *N. ceranae* used in the present study are realistic under field conditions, and both stressors were able to affect AHB health in different ways. Thus, further research is warranted to better understand the mechanisms by which these stressors affect AHB health and how bees respond to their detrimental effects.

## Figures and Tables

**Table 1:** Mean sucrose syrup consumption (µL ± SE) per honey bee/24 h during 18 days was subjected to the following treatments: Control (C), *Nosema ceranae* (1 × 10^5^ spores per bee; N), sublethal dose of thiamethoxam (1 × 10^−3^ ng/µL of syrup for 18 days; T), *N. ceranae* + thiamethoxam (NT).

Treatments	N	Mean ± SE
C	108	27.7 ± 1.3
N	108	24.6 ± 1.8
T	108	25.2 ± 1.8
NT	108	33.3 ± 6.1 F_3, 428_ = 1.376, *p* = 0.250

**Table 2:** Mean proportion (±SE) of honey bees infected with *Nosema ceranae* and spore counts at 18 days post-inoculation. Bees of both treatments were inoculated with 1 × 10^5^ spores per bee. NT treated bees were also exposed to a sublethal dose of thiamethoxam (1 × 10^−3^ ng/µL of syrup for 18 days).

Treatments	Proportion Infected Bees (Mean ± Se)	No. Spores/Bee ± Se
N	37.8 ± 10.09	4.38 × 10^6^ ± 1.31 × 10^6^
NT	42.0 ± 13.81 t = −0.24, *p* = 0.8	1.35 × 10^6^ ± 6.09 × 10^5^ W = 1242, *p* = 0.47

## Data Availability

The data presented in this study will be made available upon reasonable request from the corresponding author.
